# Cardiac Manifestations from Non-FIP1L1-PDGFR*α*-Associated Hypereosinophilic Syndrome in a 13-Year-Old African American Boy

**DOI:** 10.1155/2009/804910

**Published:** 2010-01-03

**Authors:** Cindy M. Salm, Nicole E. St. Clair, James V. Lustig, Margaret M. Samyn

**Affiliations:** ^1^Department of Medicine, Aurora Health Care, Milwaukee, WI 53213, USA; ^2^Department of Pediatrics, Children's Hospital of Wisconsin, Milwaukee, WI, USA; ^3^Department of Pediatrics, Allergy, Asthma, and Clinical Immunology, Children's Hospital of Wisconsin, Milwaukee, WI, USA; ^4^Department of Pediatrics, Cardiology, Herma Heart Center, Children's Hospital of Wisconsin, Milwaukee, WI, USA

## Abstract

Hypereosinophilic syndrome (HES) is a rare disorder typically seen in males, aged 20 to 50, with a predisposition for Caucasians. It is marked by overproduction of eosinophils (>1,500/*μ*L) and multiorgan system damage due to eosinophilic infiltration and mediator release. There are multiple variants of HES. Cardiac complications are more common in myeloproliferative HES associated with the FIP1L1-PDGFR*α* mutation. Sequelae range from acute necrosis and thrombus formation to fibrosis of the endomyocardium. We describe a young boy who presented with chest pain and dyspnea. A diagnosis of HES was made after all other etiologies of eosinophilia were excluded. Although he was found to be negative for the FIP1L1-PDGFR*α* mutation, his cardiac complications included pericardial effusion and restrictive cardiomyopathy, without myocardial necrosis. Multi-organ involvement resulted in pericarditis, pleuritis, nephritis, and dermatitis. In this paper, we review his case and discuss the known subtypes of HES, the classic cardiac complications, and available treatment strategies.

## 1. Introduction

Hypereosinophilic syndrome (HES) is a systemic disease characterized by eosinophilia and multi-organ damage. Systems involved include the hematologic, cardiovascular, cutaneous, neurologic, pulmonary, gastrointestinal, splenic, hepatic, and ocular systems. Treatment is directed at suppression of the eosinophilia and mediator release [1].

## 2. History of Present Illness

A previously healthy 13-year-old African American boy developed erythematous pruritic plaques encircling his upper arms. The lesions spread over the next 2 days to his back, chest, face, and legs. After treatment with topical steroids and antihistamines, the rash resolved, but the pruritis persisted. On day 5, the patient developed shoulder and knee arthralgias. On day 12, he developed continuous dull midline chest pains rated 8 out of 10, and dyspnea while supine. He had fevers on days 15 and 18. On day 21, he presented to an emergency department and was treated with ibuprofen. The pain persisted, and on day 28, he presented to Children's Hospital of Wisconsin Emergency Department and was admitted.

## 3. Physical Examination

Vital signs on admission were temperature 36.8°C, heart rate 100 beats/minute, blood pressure 102/68 mmHg, respiratory rate 24 breaths/minute, and pulse oximetry 99% in room air. The patient had lost 20 pounds over the past month. Edema of the hands, feet, and periorbital regions was present. Breath sounds were decreased and bibasilar rales noted. Heart rate and rhythm were regular. A cardiac rub was present. The liver was palpable 2 cm below the costal margin at the right mid-clavicular line. The remainder of the exam was normal.

## 4. Laboratory and Other Diagnostic Findings

Laboratory data included a white blood cell count of 22.4 K/uL with 72% segs, 1% bands, 6% lymphocytes, 19% eosinophils (absolute eosinophil count of 5,600/*μ*L), 1% monocytes, and 1% basophils. Some eosinophils had more than 2 lobes. Hemoglobin was 9.9 g/dL, and platelets were 446 K/uL. Electrolytes, aspartate amino transferase, amino alanine transferase, and alkaline phosphatase were normal. Creatinine was 0.9 mg/dL, and blood urea nitrogen was 8 mg/dL. Erythrocyte sedimentation rate was 48 mm/hr, and C-reactive protein was 16.5 mg/dL.

The differential diagnosis included HES, a drug reaction, parasitic or fungal infection, Churg Strauss syndrome, acute eosinophilic leukemia, lymphoma, Gleich syndrome, systemic mastocytosis, adrenal insufficiency, and eosinophilic enteritis. Since HES is a diagnosis of exclusion, the other disorders were ruled out. 

To exclude a drug reaction, ibuprofen, the patient's only medication, was held. No subsequent change in the eosinophilia occurred. Urine drug screen was not performed as clinical suspicion was low. Travel history was negative. Stool testing for ova and parasites was negative. HIV, lyme, and toxocara titers were negative. Mycoplasma polymerase chain reaction was negative. Skin biopsy revealed perivascular neutrophilic and eosinophilic infiltrate without fibrinoid necrosis of the vascular wall. Since the patient was without wheezing or history of asthma, lacked paranasal sinus abnormalities, and had normal electromyography, Churg Strauss syndrome was doubtful. Total complement (CH50), C3, and C4 levels were normal. Antidouble stranded deoxyribonucleic acid antibodies, antineutrophilic cytoplasmic antibodies, and extractable nuclear antigens panel were negative. Leukemia, lymphoma, and mastocytosis were not present as the bone marrow biopsy showed eosinophilia with some immature forms, but no tumor cells or increased mast cells. An IgM level was normal and the patient lacked any known angioedema, making Gleich syndrome unlikely. IgA, IgG, and IgE were also normal. The patient lacked any gastrointestinal symptoms, making eosinophilic enteritis unlikely. 

Radiologically, chest X-ray demonstrated a small left-sided pleural effusion. Echocardiogram revealed a small pericardial effusion. Chest CT confirmed these findings, and also showed enlarged axillary lymph nodes and bibasilar atelectasis. Ultrasound revealed mildly enlarged kidneys with hyperechogenicity of the renal cortices. Eosinophils was not present in the urine, but proteinuria and sterile pyuria were noted. Coombs test was negative. The FIP1L1-PDGFR*α* (F/P) mutation characteristic of myeloproliferative HES was negative. The CD3-CD4+ T cells characteristic of lymphoproliferative HES were not present. Serum tryptase and vitamin B12 levels were normal.

## 5. Clinical Course

The patient's dyspnea increased, and oxygen dependence developed during the first 2 days of hospitalization. Lasix was administered. When the diagnosis of HES was made, the patient was treated with solumedrol 60 mg intravenous every 6 hours. In 48 hours, the eosinophil count dropped from 7,100 cells/*μ*L to 100 cells/*μ*L. Over the following 3 weeks, the dyspnea and oxygen need gradually resolved. The boy was transitioned to prednisone 60 mg daily. 

**Figure 1:**
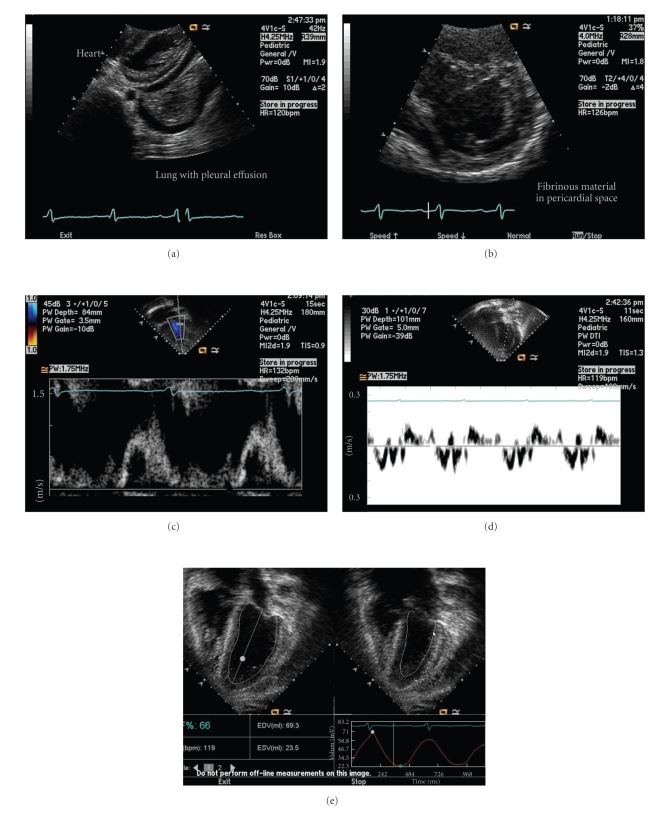
Echocardiogram showing (a) pericardial effusion and pleural effusion at presentation (from subcostal imaging plane), (b) progression of pericardial effusion with fibrinous material (from short axis plane), (c) mitral valve Doppler inflow signal with reversal of E and A waves, suggestive of restrictive myocardial disease with impaired diastolic function, (d) impaired diastolic function suggested by low Tissue Doppler signal, and (e) preserved left ventricular systolic function.

**Figure 2:**
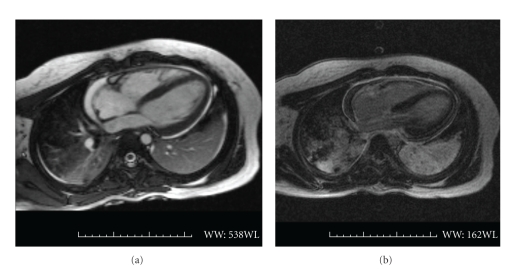
(a) Steady-state free precession cardiac MRI 4 chamber image showing small pericardial effusion, trace pleural effusion, and posterior lung field atelectasis. (b) Delayed enhancement cardiac MRI image (10 minutes postgadolinium infusion) showing no myocardial delayed enhancement; thus, no fibrosis. Visceral and parietal pericardium noted in white with pericardial effusion appearing dark between these two layers.

During the initial 4 weeks of hospitalization, serial echocardiograms showed enlargement of the pericardial effusion from small to moderate with myocardial disease with impaired diastolic function (see Figure 1). Serial cardiac enzymes remained normal. Endomyocardial fibrosis was not present on cardiac MRI (see Figure 2). On discharge, the patient had neither chest pain nor shortness of breath. The absolute eosinophil count remained normal. 

## 6. Discussion

The diagnosis of HES requires (1) eosinophilia of more than 1,500 cells/*μ*L for at least 6 months, (2) exclusion of other causes of eosinophilia, and (3) signs and symptoms of single or multiple organ involvement [1]. Revising the criteria to include patients who require treatment prior to six months of eosinophilia has been proposed [2]. HES is most commonly seen in Caucasian males aged 20 to 50 [3–5]. The exact prevalence of the disease is unknown [3]. 

Myeloproliferative Hypereosinophilic syndrome (M-HES) can be diagnosed by detecting a chromosomal aberration on chromosome 4q12 (FIP1L1-PDGFR*α* mutation), which results in constitutive tyrosine kinase activity. Patients with this mutation typically respond well to imatinib mesylate (Gleevac, Novartis), a tyrosine kinase inhibitor [3]. One in three patients who respond to imatinib mesylate lacks the F/P mutation, suggesting involvement of other unidentified tyrosine kinase genes [6, 7]. In 2004, Klion et al. considered diagnosis of M-HES when four of eight laboratory criteria listed were met in addition to the criteria that define HES: dysplastic eosinophils, increased B12, increased tryptase, anemia/thrombocytopenia, increased bone marrow cellularity with left shift, myelofibrosis, and dysplastic mast cells, or megakaryocytes in the bone marrow [8]. Cardiac manifestations appear to be most common in M-HES and range from acute necrosis to thrombus formation and fibrosis [5]. Symptoms and findings seen during the thrombotic or fibrotic stage include dyspnea, chest pain, left/right ventricular failure, and mitral/tricuspid regurgitation [3]. Late manifestations include arrhythmias and chronic heart failure. Despite the increased risk of thromboembolic disease in patients with HES, anticoagulant therapy is not formally recommended, because it has no effect on preventing further thrombosis [4]. Patients with M-HES, especially those with the F/P mutation, possess a particularly poor prognosis due to their increased risk for cardiac complications and risk of developing leukemia [7]. 

Restrictive cardiomyopathy is a rare form of cardiomyopathy, accounting for less than 3% of cardiomyopathies in children [9]. It is characterized by myocardial disease causing restricted ventricular filling (diastolic dysfunction) with preserved systolic function. Primary and secondary forms have been identified, with secondary forms more common in adults and related to infiltrative processes, including amyloidosis, anthracycline cardiomyopathy, radiation toxicity, sarcoidosis, scleroderma, storage diseases, and HES. In children, restrictive cardiomyopathy is more often idiopathic, related to anthracycline chemotherapy or endocardial fibroelastosis, with up to one third being familial [10]. Presenting features of restrictive cardiomyopathy are similar to those demonstrated by this patient with respiratory symptoms, hepatomegaly, and peripheral edema being key features. Symptoms are related to poor ventricular filling resulting from decreased ventricular compliance, which leads to elevated central venous pressures, dilated atria and, if the left ventricle is affected, pulmonary hypertension. Echocardiography allows initial evaluation of atrial size, right ventricular and pulmonary pressure, and ventricular systolic function. Doppler assessments of atrioventricular valve inflow and tissue Doppler methods permit determination of diastolic function. Using delayed enhancement pulse sequences after gadolinium injection, cardiac MRI may be useful for detection of myocardial fibrosis and rare intracardiac thrombi [11]. Cardiac MRI allows confirmation of ventricular systolic function using quantitative measures and can detect pericardial disease to rule out pericardial constriction [12]. 

In contrast to M-HES, patients with lymphoproliferative hypereosinophilic syndrome (L-HES) have less risk of developing cardiac involvement. Instead, end-organ complications of hypereosinophilia, usually consisting of cutaneous manifestations, are present [3]. L-HES is characterized by phenotypically aberrant T-cells that overproduce eosinophilopoietic cytokines, predominately IL-5, but also IL-4, IL-13, TNF-*α*, and granulocyte monocyte colony stimulating factor [7]. The most frequently reported T-cell phenotype has been CD3-CD4+, but many other variants exist. Long-term prognosis of L-HES is poor due to the high risk of developing T-cell lymphoma from malignant transformation of these aberrant T cells. There is no specific treatment for L-HES, though corticosteroids remain the cornerstone [3]. Emerging research on an anti-interleukin-5 monoclonal antibody provides hope for corticosteroid-sparing in these patients. This agent, mepolizumab, has been studied in HES patients negative for the F/P mutation, who are dependent on corticosteroids for suppression of eosinophilia. Concomitant usage of mepolizumab and corticosteroids has suppressed eosinophilia while reducing, if not eliminating, the corticosteroid requirement [7].

## 7. Followup and Conclusion

Although the patient was tapered off of prednisone over the first 3 months postdischarge, he demonstrated very poor followup and medication compliance in the subsequent 18 months. Serial pulmonary function tests continued to worsen. The last results, obtained eleven months after discharge, showed a forced vital capacity 73% of predicted, forced expiratory volume in one second 62% of predicted, and forced expiratory flow between 25% and 75% of forced vital capacity 47% of predicted. His fluticasone inhaler was increased to three times daily. At his cardiology follow-up appointment four months after discharge, he was without cardiovascular complaints, except for mild facial edema. His echocardiogram showed normal biventricular systolic function with only minimal septal bounce and no signs of restrictive cardiomyopathy or residual pericardial effusion. He was advised to continue aspirin daily and diuretics, as needed for facial swelling. He failed to attend any further cardiology appointments.

Most recently, 18 months postdischarge, the patient presented to the Emergency Department with fever, chills, and malaise. His only reported medication at this time was aspirin. Vital signs revealed a temperature 37°C, heart rate 104 beats/minute, blood pressure 122/67 mmHg, respiratory rate 36 breaths/minute, and pulse oximetry 98% in room air. Examination was unremarkable. Laboratory data included a white blood cell count of 17.1 K/uL with 54% segs, 4% bands, 15% lymphocytes, 23% eosinophils, and 4% monocytes. Electrolytes, liver function tests, troponin, and CPK-MB levels were normal. Electrocardiogram showed normal sinus rhythm with nonspecific T wave abnormalities and borderline prolonged QTc (458 msec). Chest X-ray showed normal cardiac silhouette and pulmonary vascular markings. Outpatient management with oral prednisone 30 mg twice daily for 7 days was advised. The patient failed to followup.

In this case, a young African American boy with HES developed rapid pericardial effusion and echocardiographic evidence of impaired diastolic function with suggestions of developing restrictive cardiomyopathy over one month. Cardiac MRI showed no evidence of acute myocardial necrosis. The patient also had evidence of pleuritis, nephritis, and dermatitis. The patient's cardiac manifestations, young age, and race (African American) are all atypical for the non-FIP1L1-PDGFR*α* variant of HES [3–5]. His marked response to high dose steroids was initially life-saving, with near resolution of echocardiographic changes in the immediate posthospitalization; yet, his subsequent poor followup and medication noncompliance has made his prognosis grave. 

## References

[1] Chusid MJ, Dale DC, West BC, Wolff SM (1975). The hypereosinophilic syndrome: analysis of fourteen cases with review of the literature. *Medicine*.

[2] Roufosse F, Cogan E, Goldman M (2004). Recent advances in pathogenesis and management of hypereosinophilic syndromes. *Allergy*.

[3] Sheikh J, Weller PF (2007). Clinical overview of hypereosinophilic syndromes. *Immunology and Allergy Clinics of North America*.

[4] Spry CJF (1982). The hypereosinophilic syndrome: clinical features, laboratory findings and treatment. *Allergy*.

[5] Weller PF, Bubley GJ (1994). The idiopathic hypereosinophilic syndrome. *Blood*.

[6] Cools J, DeAngelo DJ, Gotlib J (2003). A tyrosine kinase created by fusion of the PDGFRA and FIP1L1 genes as a therapeutic target of imatinib in idiopathic hypereosinophilic syndrome. *The New England Journal of Medicine*.

[7] Rothenberg ME, Klion AD, Roufosse FE (2008). Treatment of patients with the hypereosinophilic syndrome with mepolizumab. *The New England Journal of Medicine*.

[8] Klion AD, Robyn J, Akin C (2004). Molecular remission and reversal of myelofibrosis in response to imatinib mesylate treatment in patients with the myeloproliferative variant of hypereosinophilic syndrome. *Blood*.

[9] Lipshultz SE, Sleeper LA, Towbin JA (2003). The incidence of pediatric cardiomyopathy in two regions of the United States. *The New England Journal of Medicine*.

[10] Schwartz ML, Colan SD (2003). Familial restrictive cardiomyopathy with skeletal abnormalities. *American Journal of Cardiology*.

[11] Barkhausen J, Hunold P, Eggebrecht H (2002). Detection and characterization of intracardiac thrombi on MR imaging. *American Journal of Roentgenology*.

[12] Krombach GA, Saeed M, Higgins CB, Higgins CB, De Roos A (2003). Myocardial and pericardial diseases. *Cardiovascular MRI and MRA*.

